# Curious Illustrations of St. John Long's Cures of Consumption, with Obituary Attestations

**Published:** 1829-07-01

**Authors:** 


### LXI.
### Cure of Consumption. By St. John Long.

Our readers must have seen the long list of cures lately blazoned forth in the John Bull and various other newspapers, by the wonder-worker of Harley-street.

By the Post of this morning (June 15th) we received two newspapers, Bell's Weekly Messenger, and the Leeds Mercury. In the former was printed very conspicuously the letter with its testimonials (No. 1.) In the latter (Leeds Mercury) a single line dispelled the fair illusion of hope on one hand, and unprincipled mendacity on the other! Mr. St. John Long, and his puffers require some strong opiates to make them sleep with easy consciences.

(1.)

London, August, 17, 1828.

My dear Sir,—For seven years previous to placing myself under your care, I suffered great general debility, with violent cough, and expectoration, and was dreadfully emaciated. I was also afflicted with spitting of blood, pains in the chest and side, extreme difficulty in breathing, could not lie down in bed without a chair for my support, and despairing of recovery. Now, my flesh has become firm and healthy, I have appetite, can sleep, and walk five or six miles without much fatigue. c.

MARTHA HUDSON.

Hipperholm, Halifax, Yorkshire, Feb. 16, 1829.

It is some months since I left your care, and I have gone on progressively gaining health, having stood the severity of the winter months astonishingly well, though a cough still remains. The full history of my case is recorded in your book, which bears testimony of the merits of your important discovery.

Gratefully yours,

MARTHA HUDSON.

We, whose names are under-written, with pleasure attest the truth of the above statement :—

| | |
|---|---|
| Richard Hudson, M.A. Father of Martha Hudson. | Richard Hartley, D.D. Brother in-law of Martha Hudson. |

(2.)

Died.—On Saturday last, at Hipperholm, Martha, the second daughter of the Rev. Richard Hudson.

Our readers will also remember that in the 18th Number of this Journal, (Sept. 1828) page 533, we stated the case of a young man residing at Kensington, (the late Mr. Don) said to be under cure for confirmed consumption by St. John Long. If the reader will look back to the article, it will be seen that we pronounced the lungs to be sound.

A few weeks ago Mr. Don was seized with enteritis, and died in four days. Several medical gentlemen examined the body, and lo! the lungs were found perfectly sound, and no trace of tubercles or other phenomenon of phthisis in the chest!

